# Linking Processing Parameters and Rheology to Optimize Additive Manufacturing of k-Carrageenan Gel Systems

**DOI:** 10.3390/gels8080493

**Published:** 2022-08-09

**Authors:** Simona Russo Spena, Nino Grizzuti, Daniele Tammaro

**Affiliations:** Dipartimento di Ingegneria Chimica, Dei Materiali e Della Produzione Industriale, University of Naples Federico II, P.le Tecchio 80, 80125 Naples, Italy

**Keywords:** additive manufacturing, 3D printing, k-carrageenan, cooling history, rheology

## Abstract

Additive manufacturing—in particular, three-dimensional (3D) printing—has been introduced since the late 1980s, offering a novel paradigm for engineering design and manufacturing, as it allows the fabrication of very complex structures. Additive manufacturing of hydrogels is a very popular method to produce scaffolds to be used in tissue engineering and other biomedical applications, as well as in other advanced technological areas. When printing a thermoreversible physical hydrogel, a subtle balance between thermal and rheological parameters exists. The characteristic times of the sol–gel transition, regulated by a well-defined thermal history, must be optimized with respect to the characteristic processing times. In this work, we use this thermo-rheological approach to the additive manufacturing of a physical hydrogel. A low-cost desktop 3D printer for thermoplastic polymers was suitably modified to print a 1.5 wt% solution of k-carrageenan. The thermal behavior of the printer was determined by performing experimental measurements of the temperature–time evolution during the different processing steps, from solution loading, to the extrusion of the incoming gel, to the final solidification stage. In parallel, linear viscoelastic oscillatory shear measurements were performed in a rotational rheometer under thermal histories as close as possible to those previously measured in the printing process. The comparison between the rheological results and the quality of printing under different thermal histories is presented and discussed, highlighting the main relations between rheological and processing behavior, which are helpful in the assessment and optimization of the printing conditions.

## 1. Introduction

Three-dimensional (3D) printing, also known as additive manufacturing (AM) or rapid prototyping (RP), has been introduced since the late 1980s, and provides a new paradigm for engineering design and manufacturing [1,2]. AM encompasses a group of emerging technologies that create objects from the bottom up by depositing material in a layer-by-layer pattern created in pre-designed files [3]. With this technology, a complex structure, which cannot be made using traditional manufacturing methods, can be fabricated easily [4]. AM methods are in continuous development, finding widespread industrial applications in the electronics [5], automotive [6], aerospace [7], and medical engineering industries, among many others [8,9]. The key features of AM technologies are rapid prototyping, and the ability to print structures of different sizes, to reduce printing defects, and to enhance mechanical properties [10]

In the last few years, AM has been widely used in tissue engineering. Bioprinting, cell printing, and even organ printing are the labels coined for the printing of tissues using AM [11]. In 2009, Mironov et al. defined bioprinting as “the production of complex living and nonliving biological products from raw materials such as living cells, molecules, extracellular matrices, and biomaterials” [12]. Bioprinting combines 3D printing technology, cell biology, and materials science, by linking a device that enables the deposition of bio-inks with the build platform, where cooling leads to solidification [13,14]. Based on the printing mechanisms, bioprinting technologies can be divided into three groups: robotic dispensing, inkjet bioprinting, and laser-assisted bioprinting [15]. The inks mostly used as building materials for extrusion bioprinting are based on hydrogels, either in the form of gel precursors or as performed gels.

Thermoresponsive gels are materials whose mechanical strength changes abruptly across the gelatinization temperature. These systems provide a highly hydrated environment that supports the encapsulated cells, and generally offer shape retention to maintain the form of the as-printed constructs [16]. Due to their peculiarities, hydrogels formed by k-carrageenan (kC) have been the subject of numerous studies [17]; kC is extracted from red seaweeds, and is both biocompatible and biodegradable [18]. Its water solutions undergo a thermoreversible gelation, where the polymer chains extend and associate between one another, forming a three-dimensional network composed of double-helical aggregates connecting flexible chain segments [19,20]. The formation and dissociation of double helices results in a hysteresis between cooling and heating cycles, with the melting temperature being higher than the gelatinization temperature [21]. Sijun et al. studied the scaling laws of the gelation and melting temperature as a function of kC concentration by means of differential scanning calorimetry and rheology [22]. They found that the relationship was almost linear in the concentration range between 2 and 4 wt% kC.

In AM processes, the properties and composition of inks can be considered among the most important factors. A material can be used as an ink if it has appropriate flow properties for extrusion as well as being able to self-support during and after the printing process [22,23]. Many authors have investigated the 3D printing of hydrogels, with the aim to achieve high resolution and good mechanical properties of the products, trying to correlate the rheological properties of the candidate inks with 3D printing performance and the choice of parameters. Yang et al. [24] found equations that allow the choice of printing parameters—such as nozzle diameter, volumetric flow rate, and printing speed—to be optimized. However, they did not consider the role of material properties. Some researchers investigated the effects of printing parameters on the geometric dimensional accuracy of 3D-printed patterns [25]. One of the main challenges in this direction is to establish a reproducible method for the assessment of the printability of inks [26]. Werner et al. [27] investigated the viscoelastic properties of a temperature-dependent gelatin-based solution, and correlated them with the printability of the material. They found that the elastic modulus G′ must be greater than 2 kPa at the printing temperature and greater than 23 kPa at the temperature of the environment where the object is printed. Recently, Liu et al. [3,28] studied the correlation between rheological properties and 3D printability, and provided some information on 3D food printing of kC/xanthan/starch and kC/Pleurotus ostreatus multicomponent systems. Their results show that the extrudability of inks is significantly affected by yield stress and shear-thinning behavior, and that the rheological characterization of the inks can help to improve the resolution and shape retention performance.

Although the scientific literature tries to evaluate some properties (e.g., yield stress, shear thinning, recovery behavior) that thermoreversible inks must have, the 3D printing of thermoresponsive gel inks is still challenging, as it requires expensive and time-consuming trial-and-error processes to obtain the desired structures. To enable the successful printing of such materials, the study of the dynamics of the sol–gel transition is particularly relevant, as it determines both the extrusion behavior when in the storage unit of the printer and the post-printing self-support behavior [29,30,31].

Despite the high number of papers published on bioprinting of hydrogels [32,33,34,35,36,37], the cooling history during the 3D printing process of hydrogels has not yet been investigated in detail [38,39,40,41]. In this work, for the first time, the modelling of the complex thermal history of hydrogels, along with its link to the gelation kinetics and the design of a reproducible printing procedure, is considered. The aim of the present work is twofold: On the one hand, we connect the 3D printability to the rheological response of the hydrogel through a detailed investigation of the cooling history during the extrusion. On the other hand, we investigate the effects of the printing parameters of a specific 3D printer on the printability of k-carrageenan. Based on experiments, we determine the relationships between the relevant printing parameters—such as volumetric flow rate and temperature—and the printing quality of the expected structures. The 1D and 3D structures are optically characterized and compared, in order to contribute to the development of a design-oriented tool for future bioprinting processes.

## 2. Materials and Methods

K-carrageenan (CAS 11114-20-8) and bi-distilled water were purchased from Sigma-Aldrich (Germany) and used as received. Solutions of kC at a weight concentration of 1.5% were prepared by dissolving the required amount of polymer in water at 80 °C with the help of a heated magnetic stirrer at 300 rpm for 2 h. On the one hand, the kC concentration must be high enough to allow for gel formation upon cooling; the limiting concentration for gelling is about 0.5% [30]. On the other hand, the gelation temperature, which is an increasing function of concentration, must be low enough to be compatible with the cells’ proliferation. For the above reasons, we fixed the concentration of our model solution to 1.5 wt%, without loss of generality. The obtained solution, divided into 22 mL vials, was rapidly cooled to 5 °C in 2 min, and then stored in the refrigerator. This preparation allowed us to slow down the sample degradation. Before each test, the sample was heated to 70 °C for 20 min to cancel any memory effect of the previous deformation history. 

The rheological behavior of the bio-ink formulations was measured using a controlled stress rheometer (MCR-702, Anton Paar, Linz, Austria) equipped with a parallel-plate geometry (diameter 50 mm, gap 1 mm). Oscillatory shear measurements were always conducted within the linear viscoelastic regime. Temperature control was guaranteed by a Peltier unit, able to perform isothermal as well as temperature ramp experiments with an accuracy of ±0.1 °C. All rheological experiments were performed at least in triplicate. Good reproducibility was always found. For this reason, this aspect was not be considered further.

**Figure 1:**
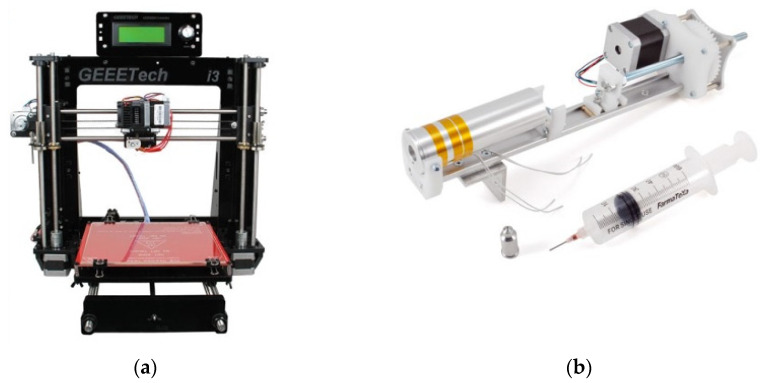
The hydrogel printing system: (**a**) the original thermoplastic printer; (**b**) the fluid extrusion add-on.

3D printing experiments were performed using a Prusa I3 Pro B printer (GeeeTech, Wuhan, China). The printer (Figure 1a), conceived for fused-filament thermoplastic polymers, was suitably modified for use with hydrogels by replacing the original injection system with a fluid extruder, as shown in Figure 1b. The extruder (Choco 3Drag extruder set, Futura Group, Verona, Italy) is characterized by a cylindrical aluminum body with an outer and inner diameter of 35 mm and 31.6 mm, respectively, which hosts a heating resistance. A 60 mL plastic syringe ending with a 0.9 mm internal diameter needle was loaded with the bio-ink and placed inside the aluminum cylinder. The syringe piston was driven by a Nema17 step motor. The printer, including the piston actuator and the heating system, was computer-controlled using the free software EasyPrint 3D (GeeeTech, Wuhan, China).

The printability of the bio-inks was evaluated based on the consistency of single rectilinear filaments and square-shaped walls. Images and videos of the printed constructs were captured using a 64-megapixel smartphone camera (Xiaomi Redmi Note 9 Pro, Wuhan, China). A ruler was used to perform the pixel-to-length conversion. Quantitative dimensional analysis was then performed by using the ImageJ (National Institutes of Health and the Laboratory for Optical and Computational Instrumentation, Boston, MA, USA) free software.

## 3. Results and Discussion

### 3.1. Characterization of the 3D Printer’s Thermal History

The 3D printing process of a thermoreversible hydrogel can be divided into several steps, each corresponding to a specific thermal condition [3]. In the loading stage the bio-ink is in the sol state at a relatively high temperature, so that it can be loaded into a syringe and prepared for printing. In the subsequent extrusion stage, the temperature is lowered to a suitable value close to the gel point. The choice of this intermediate temperature is particularly crucial, as the material must be sufficiently fluid to be extruded through the syringe nozzle, and must also quickly undergo the sol–gel transition to transform into a dimensionally stable filament. In the third, self-supporting stage, the filament—now gelled—is deposited on top of the growing construct, and its temperature reaches the final, lowest value.

In order to relate the rheological behavior of the ink to the printing process performance, the same thermal history should be reproduced in the rheometer. To this end, preliminary experiments were devoted to the measurement of the temperature–time evolution in the printer. It must be noted that since the extrusion systems was equipped only with a heating system, the cooling history could not be controlled, and was essentially based on the natural convection heat transfer between the fluid and the exterior.

The temperature evolution of the sample in the printer process was monitored in two independent steps: In the first experiment, the temperature difference between the loading and extrusion stages was measured by means of a thermocouple embedded in the bulk of the syringe containing the solution. The kC solution was loaded into the extruder at the initial temperature of 70 °C, to erase the possible presence of gel-like structures. The syringe temperature was kept constant at a lower value, thus simulating the cooling-down stage from the loading temperature to the printing temperature. Extruder temperatures between 28 and 40 °C—that is, around the onset of the sol–gel transition (see the rheological measurements below)—were imposed.

In a second experiment, after the kC ink had reached the stationary extruder temperature, a small droplet of about 4 mm in diameter was generated by the printer and deposited onto the printer plate. In this case, the droplet temperature was measured by means of a thermocouple placed and maintained in its center, whereas another temperature sensor was glued to the printer plate, which was kept at room temperature, thus simulating the self-supporting stage of the printing process.

**Figure 2:**
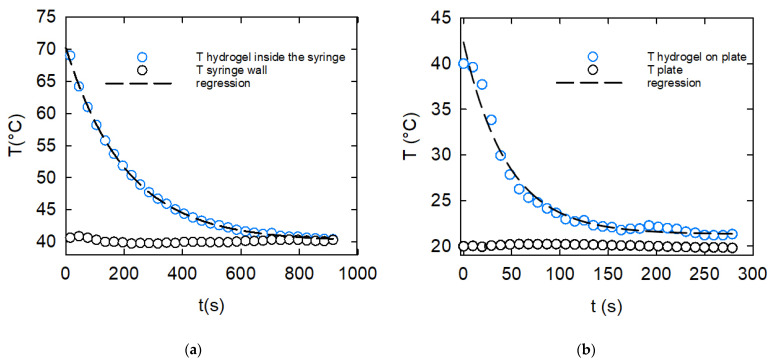
The kC temperature as a function of time during the printing stages: (**a**) cooling down from the loading temperature to the extruding temperature (the experiment at 40 °C is shown); (**b**) cooling down of the generated droplet (see text) after extrusion, during the self-supporting stage. The droplet was printed at 40 °C on the printer plate kept at 25 °C. Lines correspond to the best fit of the data to Equation (1).

The results of the two cooling experiments are shown in Figure 2a for the extruder stage, and in Figure 2b for the self-supporting stage. Both temperatures followed an exponential decay of the following type:

(1) $$T\left(t\right)=T_{\infty }+\left(T_{0}-T_{\infty }\right)exp^{\left(-t/\tau \right)}$$

where $T_{0}$ and $T_{\infty }$ are the initial and long-term equilibrium temperature in each experiment, respectively, and τ is a decay characteristic time. The solid lines in Figure 2 are the best fit of Equation (1) to the experimental data, and confirm the good agreement between the experiments and the exponential behavior. The regression values of the three parameters for each case are reported in Table 1.

Both Figure 2 and Table 1 clearly indicate that the cooling step from the loading temperature to the extruding temperature was much faster than the subsequent phase of cooling to room temperature. In particular, the average cooling rate of the first step was in the order of 1 °C/min, whereas that of the material cooling after extrusion was in the order of 5 °C/min.

Before concluding this section, it is important to note that the cooling dynamics of the gel from the extruding temperature to the final solidification temperature were observed using a droplet of 4 mm diameter. However, in the real printing operation, the material is extruded in the form of a filament of about 0.9 mm diameter. The use of a droplet of larger size is dictated by the necessity of measuring the temperature evolution inside the material. Due to the finite size of the thermocouple head, this would have been impossible for the thin extruded filament. As a consequence, the characteristic time of the self-supporting stage is expected to be smaller, and must be recalculated. This can be done by modelling the heat transfer process during cooling, as described below.

We first evaluated the Biot number (Bi), for the heat exchange process between the sphere and the quiescent surrounding air. *Bi* gives a direct indication of the relative importance of conduction and convection at the surface of a body during cooling or heating. In this case, Bi is given as follows:

(2) $$Bi=\frac{2k_{AIR}}{k_{s}}$$

where $k_{AIR}$ and $k_{s}$ are the thermal conductivity of the air and of the sphere, respectively. The latter is assumed to be equal to that of water. Since $k_{AIR}=0.026\;W/\mathrm{mK}$ and $k_{s}=0.63\;W/\mathrm{mK}$, one has Bi≅0.08≪1, and the droplet temperature can be considered to be uniform. In this case, the energy balance for the sphere can be written as follows:

(3) $$\frac{\pi D_{s}^{3}\rho_{GEL}C_{GEL}}{6}\frac{dT_{GEL}}{dt}=\pi D_{s}2k_{AIR}\left(T_{AIR}-T_{GEL}\right)$$

where $T_{GEL}$ is the time-variable sphere temperature, $D_{s}$ is its diameter, and $\rho_{GEL}$ and $C_{GEL}$ are the density and specific heat, respectively. As for the thermal conductivity, its values are approximated to those of water. $T_{AIR}$ is the constant room temperature. 

Equation (3) is solved with the initial condition $T_{GEL}=T_{extr}\;at\;t=0$, where $T_{extr}$ is the extrusion temperature, giving:

(4) $$T_{GEL}\left(t\right)=T_{AIR}+\left(T_{extr}-T_{AIR}\right)exp^{\left(-t/\tau_{s}\right)}$$

where $\tau_{s}$ is the characteristic cooling time for the sphere:

(5) $$\tau_{s}=\frac{D_{s}^{2}\rho_{GEL}C_{GEL}}{12k_{AIR}}$$

Assigning numerical values to the physical parameters in Equation (5) yields:

(6) $$\tau_{s}=\frac{D_{s}^{2}\rho_{gel}C_{gel}}{12k_{AIR}}≅215\;s=3.6\;\min$$

The value of $\tau_{s}$ obtained from the model compares reasonably well with the one derived from the experimental measurements and reported in Table 1, confirming the validity of the above analysis. The smaller value (a factor of about two) obtained by the model can be justified by considering that in the real measurement heat is also dissipated at the interface between the drop and the printer plate—something that is not considered in the model.

We are now in a position to estimate the characteristic time required for the filament cooling. The same analysis shown above can be carried out for a cylindrical filament of diameter $D_{f}$. In this case, the solution for the filament temperature has the same form as Equation (4), with a characteristic time given as follows:

(7) $$\tau_{f}=\frac{D_{f}^{2}\rho_{GEL}C_{GEL}}{8k_{AIR}}=\left(\frac{3}{2}\frac{D_{f}^{2}}{D_{s}^{2}}\right)\tau_{s}$$

Equation (7) shows, as expected, that the characteristic cooling time of the 0.9 mm diameter filament is much smaller than that of the 4 mm diameter sphere. Using the value of τ for experiment (b) in Table 1, one has $\tau_{f}=0.076\tau_{s}≅4\;s$. This value is compared in a subsequent section to the gelation time measured by rheology.

### 3.2. Rheological Measurements and Optimization of the Printing Process Parameters

As discussed above, each stage of the printing process is characterized by a well-defined thermal history. This, in turn, is expected to significantly affect the quality and the final properties of the printed construct. In this section, we aim to show how rheological measurements can be used to optimize the choice of the printing process parameters.

#### 3.2.1. Extrusion Temperature

**Figure 3:**
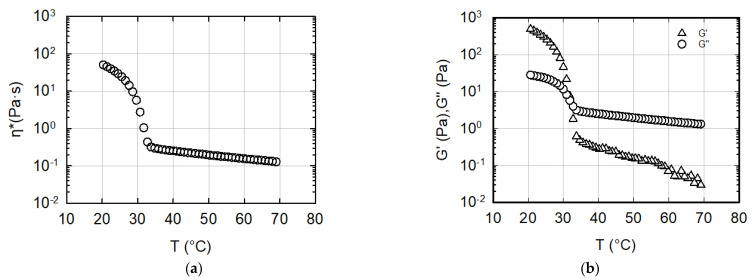
The linear viscoelastic response of the kC bio-ink to a cooling-down history from 70 to 10 °C at a rate of 1 °C/min; the frequency is 10 rad/s, and the strain is 0.5%: (**a**) complex viscosity; (**b**) elastic and loss moduli.

Figure 3 shows the classical rheological response of the kC ink during the sol–gel transition. The sample was loaded into the rheometer at a temperature of 70 °C, and was then cooled down at a cooling rate of 1 °C/min. An oscillatory shear flow was simultaneously applied, allowing for the monitoring of the sol–gel transition. As widely reported in the literature, the sol–gel temperature value depends on a number of parameters, such as biopolymer concentration [31], presence of co-solute [37,38], and heating/cooling rate [39]. In this test, a rate of 1 °C/min was chosen in order to obtain as accurate a value as possible. This temperature value made it possible to individuate a range in which the printing tests could be concentrated.

The fingerprint of the transition appears very clearly in Figure 3 as the sudden, dramatic increase in the viscoelastic moduli below a critical temperature. It is worth noting that the sample shows a Newtonian rheological behavior at the loading temperature of 70 °C. This Newtonian behavior at 70 °C was confirmed by steady-state measurements in Couette geometry, which are not reported here for brevity. The corner point of Figure 3a, where the complex viscosity is reported, indicates that the sol–gel transition begins at a temperature of about 36 °C. At a slightly lower temperature (about 32 °C, see Figure 3b), the elastic modulus overcomes the loss modulus, suggesting that the material changes its behavior from liquid-like to solid-like.

The data in Figure 3 determine a first, rheology-related process indication, as far as the extrusion temperature is concerned. According to the kC’s viscoelastic response upon cooling, an excessively high extrusion temperature would result in the production of a purely viscous, low-viscosity, structureless filament, with obvious negative consequences on the structural consistency of the construct. Conversely, an excessively low temperature would shift the kC material into the solid-like region, thus making the generation of a smooth, continuous filament impossible.

**Figure 4:**
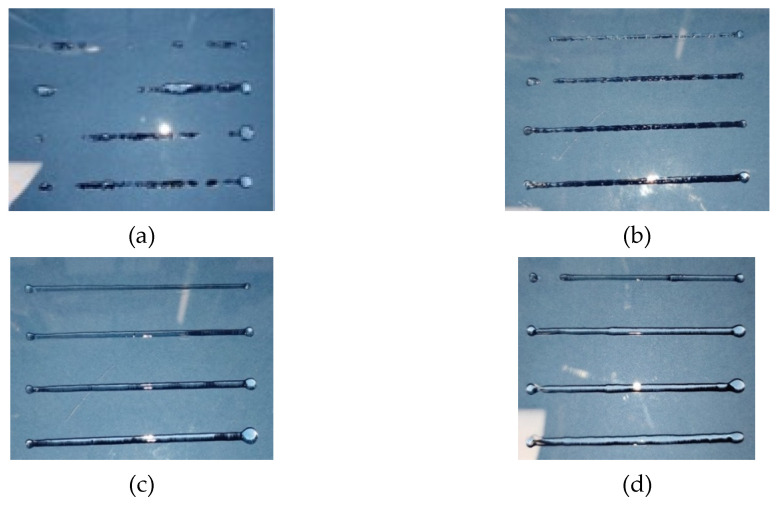
Snapshots of filaments printed at different extrusion temperatures and volumetric flow rates—(**a**) 28 °C; (**b**) 32 °C; (**c**) 36 °C; (**d**) 40 °C—and at a fixed printing speed of 12 mm/s.

The above considerations are confirmed by the actual printing experiments, as reported in Figure 4. The bio-ink shows three different types of printing behavior at different extrusion temperatures and volumetric flow rates, and at a fixed printing speed of 12 mm/s. When the ink was printed at a high temperature (40 °C, Figure 4d), it resulted in a swollen strand. In contrast, when the ink was fabricated at lower temperatures (Figure 4a,b; 28 and 32 °C, respectively), irregular filaments with a rough surface were produced. The ink deposited at an extrusion temperature of 36 °C showed the best result in terms of a smooth, uniform, and constant diameter shape.

**Figure 5:**
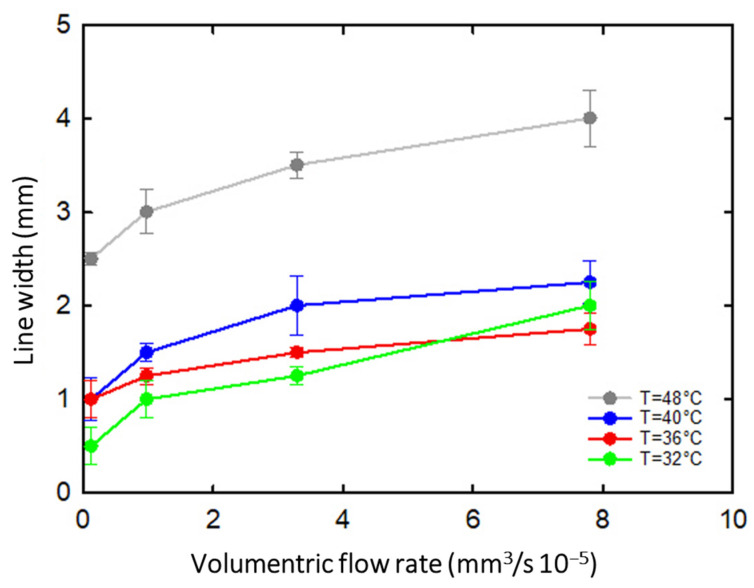
The printed filament diameter as a function of the volumetric flow rate at different extrusion temperatures; lines are intended to guide the eye.

The results of Figure 4 are further confirmed in Figure 5, where the average diameter of the printed filament is plotted as a function of the flow rate at different temperatures. The data at 28 °C are not reported because, as shown in Figure 4, an intermittent filament was produced at that temperature. Only filaments produced at a temperature between 32 and 36 °C maintained a size close to that of the 0.9 mm nozzle diameter. The effect of the flow rate, the increase in which obviously determines an increase in the filament diameter, should also be noted. However, the effect of flow rate is not addressed in the present study.

Based on the comparison between rheology and printing tests, it can be concluded that the optimal extruder temperature for printing is close to the temperature marking the onset of the sol–gel transition—in this case, 36 °C.

#### 3.2.2. Extrusion and Printing Times

Once the extrusion temperature is optimized, another important choice concerns the processing times. The printing process contains at least three relevant characteristic times. The first is the loading time—that is, the time required to load the sample at a high temperature and subsequently cool it down to the extrusion and printing temperature. Such a characteristic time is dictated by the intrinsic cooling dynamics of the printer, and was previously discussed in Section 3.1. In the specific case considered here, this time was in the order of 7 min.

A second characteristic time is the time interval for printing. Once the extrusion temperature is reached, the printer must be able to deposit a filament for a sufficiently long time to complete the printing operations. During such a time, the rheological properties of the material must remain as constant as possible, in order to guarantee the homogeneity of the construct. Obviously, the printing time is strictly related to the extrusion temperature.

The third characteristic time is the time required for the deposition of two consecutive filament layers. If the deposition time is too short, the freshly deposited filament will not possess a sufficient consistency, and is expected to lose its dimension and shape due to viscous flow. Conversely, if the time is too long, the filament will become more rigid, and a good adhesion to the previous layer will not be guaranteed. Moreover, this time will be related to the thermal history and the rheological response of the material.

**Figure 6:**
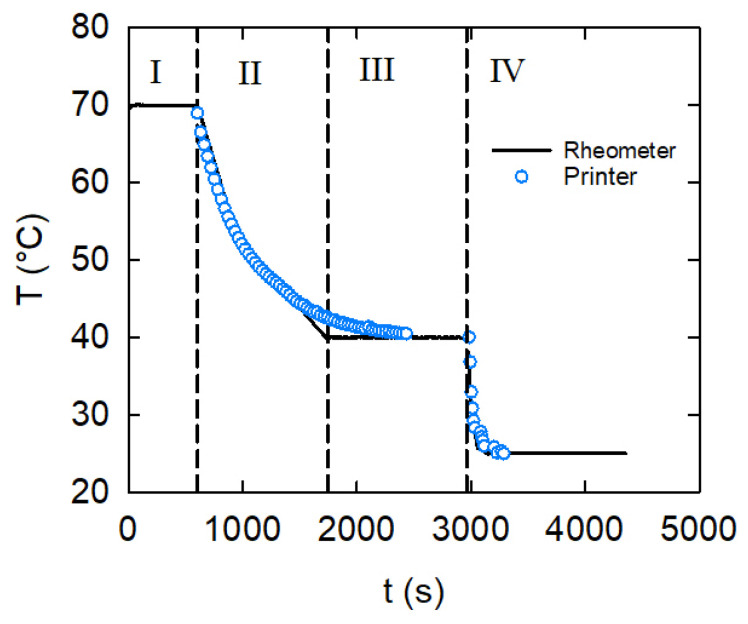
The thermal history applied to the kC ink in the rheometer simulation of the printing process. The extrusion temperature was 40 °C. The four temperature regions are discussed in the text. The measured printer temperatures, already reported in Figure 2, are added here for comparison.

In order to directly relate the printing times to the material mechanical evolution, a series of rheological tests were performed by applying the same thermal history that was measured in the different stages of the 3D printing process and reported in Section 3.1. Figure 6 shows the temperature cooling profile applied to the bio-ink, which can be divided into four main zones. In Region I, the liquid kC solution is loaded at 70 °C and allowed to rest and stabilize. Then (Region II), a first cooling is applied in order to mimic the cooling stage inside the syringe down to the extrusion temperature. The first cooling stage is terminated at the extrusion temperature value (in this case, 40 °C), followed by a constant temperature resting time interval (Region III) that simulates the printing filament deposition process. Finally (Region IV), the temperature is lowered again—this time at a faster cooling rate—thus mimicking the self-supporting stage, with the filament being cooled and allowed to stabilize at the deposition plate temperature. 

In the same figure, the experimental data collected on the printer and already presented in Figure 2 are shown. The good superposition between the rheometer and the printing thermal histories can be appreciated. In order to achieve this, and since the rheometer can only apply constant cooling rates, a piecewise sequence of cooling steps at different rates was applied. In this way, a sufficiently close correspondence between the temperature histories of the material in the rheometer and in the printer was obtained.

**Figure 7:**
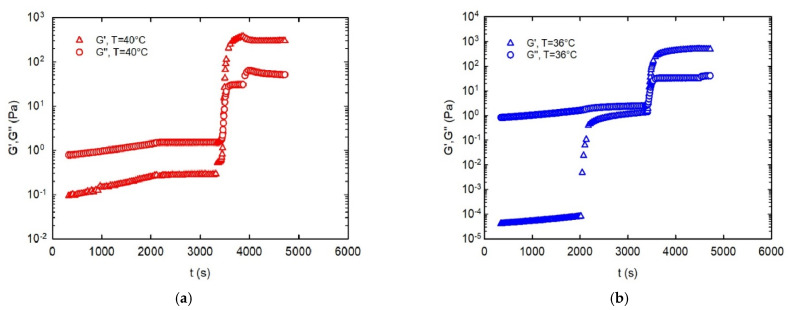

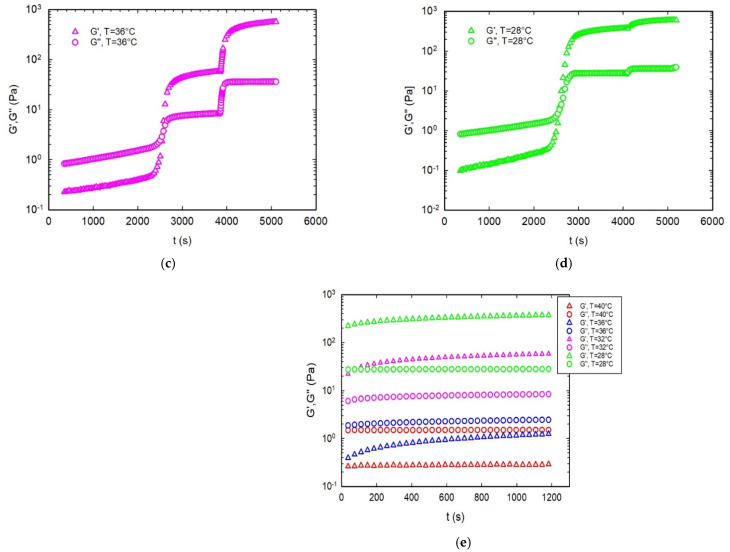
The complex modulus of the kC bio-ink as a function of time for the thermal history reported in Figure 6, and for different extrusion temperatures: (**a**–**d**) The total temperature history; the four regions correspond to the thermal changes reported in Figure 6 (see text for details). (**e**) A close-up of region III.

Figure 7a shows the rheological response of the kC samples under the thermal history reported in Figure 6. The results are reported for four extrusion temperatures, namely, 28, 32, 36, and 40 °C. For the sake of clarity, only the complex modulus is plotted.

Figure 7 shows that, for each temperature region, the same qualitative behavior is observed for all extrusion temperatures. The G’ and G’’ evolutions in Regions I and II are essentially identical, differing only slightly due to the corresponding small differences in the final temperature at the end of Region II. From that point on, however, the quantitative behavior is different, and is a strong function of the extrusion temperature. For the highest temperature (40 °C), no changes in G’ and G’’ are observed along Region III. In this case, the sample is still in the liquid state, and no transition occurs. At 36 °C, the complex modulus is seen to slowly increase during the resting time of Region III, suggesting that the ink is still extrudable, but is characterized by a higher consistency, related to the incoming sol–gel transition. The latter, however, only takes place in Region IV, where the jump in the complex modulus spans almost three orders of magnitude. The situation is completely different at the two lowest temperatures—that is, 32 and 28 °C. Here, the transition already takes place intensively in Region III, indicating that the sample changes from a liquid-like to a gel-like behavior during the extrusion stage, where the transition takes place (to a larger or smaller extent) well before the beginning of the self-supporting stage.

The rheological measurements rationalize and complete the observations already made in Section 3.1. As far as the extrusion temperature is concerned, the rheological response confirms that the optimal temperature in this case study was around 36 °C. For higher temperatures, the liquid remained liquid, whereas for lower temperatures the transition to gel was already present during the printing stage. In both cases, as already shown in the images of Figure 4, the quality of the printed filament was not acceptable.

**Figure 8:**
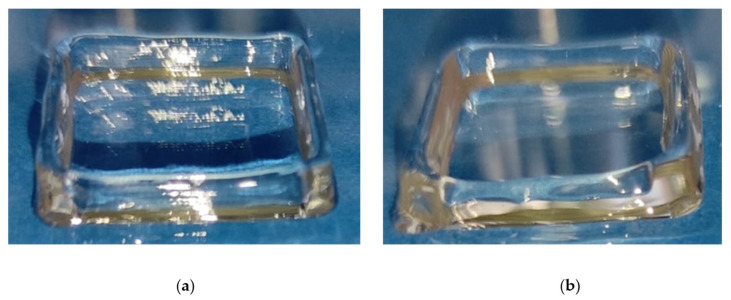
A 3D-printed square-shaped wall. The square side is 20 mm and is composed of 8 layers. (**a**) 36 °C; (**b**) 40 °C.

A closer inspection of Figure 7a–d, and of the corresponding close-up of Region III in Figure 7e, also shows that the optimal temperature of 36 °C produces a sufficiently stable material throughout the whole extrusion time. In other words, for a time window of at least 20 min (i.e., the extent of the constant-temperature Region III), the mechanical properties of the kC ink remain stable. Therefore, a uniform printing quality is expected for all layers of the construct. This point is confirmed by the image shown in Figure 8, where a squared wall printed at 36 and 40 °C is shown. The total printing time of this construct was about six seconds for each layer. Only at the former temperature was a good dimensional stability observed. No constructs printed at lower temperatures are shown because, due to the premature sol–gel transition, their filament continuity was compromised (see also Figure 2).

A final relevant piece of information comes from the rheological response in the last thermal region, corresponding to the self-supporting stage. Here, as mentioned above, the mechanical properties of the filament play a crucial role in terms of adhesion between layers. In this respect, three characteristic times are present: (i) a characteristic time for the sol–gel transition, $\tau_{S-G}$; (ii) a characteristic time for the filament cooling process (a discussed in Section 3.1), $\tau_{f}$; and (iii) a characteristic time related to the speed of printing, $\tau_{p}$. The latter can be defined as the time necessary for the consecutive deposition of two filaments on the same point of the construct. While $\tau_{p}$ is a tunable process parameter, $\tau_{S-G}$ and $\tau_{f}$ are determined by the combination of the intrinsic cooling dynamics of the printer (in our case, non-controllable) and the intrinsic sol–gel dynamics of the sample. 

Due to the non-instantaneous sol–gel transition kinetics, we expect that $\tau_{f}<\tau_{S-G}$. This is confirmed by Figure 7, where the elastic and loss moduli of kC are plotted as a function of time in the self-supporting stage (Region IV of Figure 7). Assuming that the sol–gel transition takes place at the crossover point between the moduli, we get $\tau_{S-G}≅30\;s$. It should be recalled (Section 3.1) that $\tau_{f}$ is in the order of 5 s, confirming the above inequality. It must be noted that $\tau_{f}$ and $\tau_{S-G}$ are obviously related, as faster cooling determines faster sol–gel transition kinetics. For example, different values of $\tau_{S-G}$ could be attained by changing the heat transfer conditions.

Based on the above considerations, we expect that an optimal compromise between adhesion and dimensional stability is obtained when $\tau_{f}<\tau_{p}<\tau_{S-G}$. This inequality should guarantee that the filament printing is slow enough to move towards the sol–gel transition, albeit remaining in a liquid-controlled condition (i.e., acceptable consistency, high adhesion). The validity of this hypothesis is confirmed by Video S1 in the Supplementary Materials. The printing was performed at the same extrusion temperature of 36 °C. On the left-hand side, a printing time $\tau_{p}≅40\;s\gg \tau_{SOL-GEL}$ was used, whereas on the right-hand side, $\tau_{p}≅6.6\;s\ll \tau_{SOL-GEL}$. The different degrees of adhesion, tested with the help of a pair of tweezers, can be clearly appreciated. 

## 4. Conclusions

The main scope of this work is to present a methodological approach to relate the printing performance of a thermoreversible hydrogel to its rheological behavior. The first relevant conclusion is that the material’s rheology and the 3D printing device’s characteristics are both essential and inseparable aspects to be considered to obtain an optimal printed product.

The printing parameters must always be compared and interconnected with the rheological response of the material. In particular, the present work highlights the necessity of evaluating and comparing the material’s thermal characteristic times and temperatures with those governing the printing process. Relevant rheological parameters include the temperatures marking the onset and the solid-like transition of the sol–gel evolution. The former determines the choice of the printer extrusion temperature, whereas the latter, along with the corresponding transition characteristic times, dictates the choice of the parameters governing the filament deposition process, such as the flow rate and, even more crucially, the rate of single layer deployment.

When the above considerations were applied to the specific hydrogel material (i.e., 1.5 wt% k-carrageenan aqueous solution) and the specific printer (i.e., Prusa I3 Pro B equipped with a Choco 3Drag extruder) used in this work, we found an optimal extruding temperature of 36 °C and a single-layer characteristic deposition time of about 5 seconds.

Naturally, the analysis performed in this work is not at all exhaustive, as the material and printing parameters considered are not the only ones. It should be noted that this study was carried out using a gel system based on k-carrageenan at the single concentration of 1.5%. For example, once the chemistry (i.e., the basic polymer of the bio-ink) is established, parameters such as concentration, molecular weight, and the presence of salts are all relevant in view of the optimization of the printing process, while also depending on the specific characteristics of the constructs to be printed. It is also important to underline that, in this study, the thermal evolution and the material properties related to it were the main considerations. Aspects such as the variation in the shear rate during extrusion and the microstructural changes of the material connected to it were neglected.

## Figures and Tables

**Table 1:** Cooling rate and regression parameter values.

Experiment	$T_{\infty }\;({}^{\circ}C)$	$\left(T_{0}-T_{\infty }\right)\;({}^{\circ}C)$	τ (min)
(a)	40.1	30.2	6.6
(b)	24.3	21.0	0.76

## Data Availability

Not applicable.

## Supplementary Materials

The following supporting information can be downloaded at: https://www.mdpi.com/article/10.3390/gels8080493/s1, 3D-printed square-shaped wall printed at an extrusion temperature of 36 °C: (Video S1) $\tau_{p}≅40\;s\gg \tau_{SOL-GEL}$.
